# Beyond frontiers: On invasive alien mosquito species in America and Europe

**DOI:** 10.1371/journal.pntd.0007864

**Published:** 2020-01-09

**Authors:** André B. B. Wilke, Giovanni Benelli, John C. Beier

**Affiliations:** 1 Department of Public Health Sciences, Miller School of Medicine, University of Miami, Miami, Florida, United States of America; 2 Department of Agriculture, Food and Environment, University of Pisa, via del Borghetto, Pisa, Italy; University of Colorado Health Sciences Center, UNITED STATES

## Invasive mosquitoes are more likely to adapt to urban environments

Mosquito vector species (Diptera: Culicidae) are responsible for the transmission of many pathogens and parasites to humans and animals. Current predictions indicate that more than half of the human population on the planet is at risk of vector-borne infections [1].

Most of the mosquito vectors responsible for transmitting diseases are invasive species [2]. Invasive mosquitoes of epidemiological importance, such as *Aedes aegypti* (L.), *A*. *albopictus* (Skuse), and *Culex quinquefasciatus* Say, are more likely to adapt and thrive in urban environments in low-latitude parts of the world in comparison with native species [3,4]. Moreover, invasive species often benefit from biotic homogenization processes and from the reduction in overall biodiversity by being able to increase their range and abundance in the process [3,5–7]. The substantial increase in the incidence of vector-borne diseases can be partially attributed to these factors [2].

In this viewpoint paper, we focus on two highly invasive alien mosquito species: *A*. *albopictus* in Europe and *C*. *coronator* Dyar and Knab in America, shedding light on key biological, ecological, and epidemiological issues urgently needing further attention at the forefront of vector biology and control research.

## *C*. *coronator* conquers the American continent

The invasive mosquito species *C*. *coronator* is a primary vector of Saint Louis encephalitis and West Nile viruses [8] and is increasingly becoming of public health concern in the Americas. *C*. *coronator* is a Neotropical species native from Trinidad and Tobago, and since its first description at the beginning of the 20th century, it has now been reported from Argentina to the United States.

**Fig 1:**
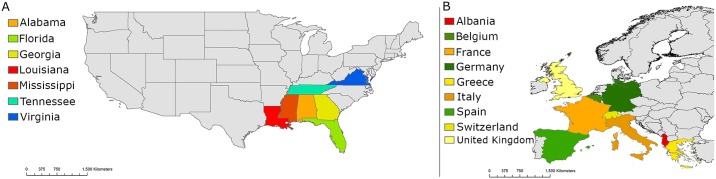
Invasion timeline of *Culex coronator* in the US and *Aedes albopictus* in Europe. The color gradient represents the patterns of expansion ranging from red (first detection), to orange and yellow (subsequent detections), to green (most recent establishment) and blue (most recent detection) for *C*. *coronator* in the US (A) and *A*. *albopictus* in Europe (B). The map was created with ArcGIS 10.2 (Esri, Redlands, California) using layers freely available at https://www.census.gov/programs-surveys/geography.html and https://www.eea.europa.eu/data-and-maps/data/external/gis-data-for-europe.

*C*. *coronator* is currently considered established in many states of the US, being first detected in Louisiana in 2004 [9]. Since then, it has spread and has been commonly found in most of the Southeastern states, including Mississippi, Alabama, Georgia, and Florida [10]. In Miami-Dade County (Florida) alone, 26,825 female *C*. *coronator* were collected in urban areas from May 2016 to November 2018 [6]. In 2016, *C*. *coronator* was detected in Tennessee, where it is currently considered an established species, and in Virginia, being the northernmost record in the US (Fig 1A) [11,12].

## *A albopictus* invades Europe

*A*. *albopictus* is thought to have dispersed together with humans throughout commercial routes from Southeast Asia to the Indian Ocean during the 18th and 19th centuries and more recently by the world trade of used tires [7,13]. Since then, *A*. *albopictus* has become, alongside with *A*. *aegypti*, the most important mosquito vector of arbovirus in the world, serving as a primary vector of chikungunya, dengue, yellow fever, and Zika viruses [7,14].

*A*. *albopictus* has recently reached and is successfully established in several Western European countries due to its vast physiological and ecological plasticity. It was first detected in 1979 in Albania, and thenceforth it was detected and is currently considered an established species in Italy since 1990, then in France since 1999, Greece and Switzerland since 2003, and Belgium and Spain since 2004 [15]. In 2015, *A*. *albopictus* was detected in Germany and successfully overwintered to 2016 [16], and in 2016, it was detected in southern England [17] (Fig 1B).

There are many other potentially dangerous neglected invasive species with only limited information available on their ecology, behavior, and vector competence. *A*. *albopictus* has most likely invaded the US in the state of Texas in 1985. Since then, it has spread throughout the Southeast, South Central, and Mid-Atlantic US and has been detected in 36 states. Its presence has also been reported in the states of Indiana, New Hampshire, and New York [18]. Results from a recent study indicate that *C*. *panocossa* Dyar has been recently introduced to Florida, probably from Cuba, and is now well established in the southern part of Miami-Dade County [19]. *A*. *atropalpus* (Coquilett), native from North America, was recently detected in Italy, France, and the Netherlands. Its dispersion was accelerated by the commercial transport of used tires and international trade [20,21]. These are just two examples of the spread of invasive mosquito species, but there are many more undetected and neglected potentially dangerous invasive species that need attention and should be monitored.

## Mechanisms employed on the invasion, establishment, and colonization of new areas by alien mosquitoes

The dispersion, establishment, and colonization of new areas by alien mosquito vectors are well documented. Many studies described and analyzed mosquito dispersal to unveil patterns and trends of expansion and colonization of new areas. However, it is currently unknown what mechanisms are employed by invasive mosquito species to disperse, establish, and thrive in urban environments. Moreover, environmental and physiological features that trigger and support mosquito dispersal are also yet to be identified.

Anthropogenic land use and land cover transformation and the subsequent increase in human population densities in large cities are important drivers for the demographic expansion of *A*. *aegypti* in a microgeographic scale [22], but there is a lack in understanding of how mosquito vectors are adapting to urban environments to make it possible for them to prosper and increase their range and abundance. Furthermore, the role of preselected insecticide resistance alleles in the success of a given invasive species to adapt to their new environments is currently overlooked and may impact their spreading, establishment, and genetic variability [23].

Importantly, there are no contingency plans or specific guidelines to prevent the invasion, establishment, and colonization of new areas by mosquito vectors. In recent years, the spread of *C*. *coronator* in the American continent and *A*. *albopictus* in the European continent make it clear that new and focused mosquito control strategies are needed to face this increasing threat (Box 1).

**Box 1. Suggested framework for future research on the development of control strategies to prevent the invasion, establishment, and colonization of new areas by alien mosquito vectors**

- Development of basic information on how and why invasive vector species are becoming locally abundant in urban habitats.
- Development of profiles of basic ecology and behavior for new and neglected invasive mosquito species to clarify their potential importance as vectors.
- Increase in awareness about the potential presence of invasive mosquito species to avoid their underestimation or even failure in detecting them due to misidentification of specimens or surveillance strategies disregarding their habitats.
- Assessment of current and future risks to determine if some invasive species should be considered a higher priority for vector control operations.

In our opinion, the adaptation of populations of alien mosquito vectors to thrive in urban environments with reduced biodiversity alongside humans represents a significant selective advantage due to the limited presence of natural predators and greater availability of artificial breeding sites and human hosts for blood feeding. Furthermore, we are experiencing an alarming deterioration of natural environments responsible for causing an environmental disequilibrium and biodiversity loss of unprecedented proportions. The worsening of the ecological imbalance caused by anthropogenic alterations in the environment will positively impact the range and abundance of mosquito vectors and, consequently, the incidence of vector-borne diseases.

## Concluding remarks and future research challenges

There is a paucity of knowledge about the mechanisms employed by invasive species to adjust their ecology and behavior in response to the different environments, and there are no effective contingency plans to guide mosquito control operations to deal with this increasing threat. Furthermore, the lack in awareness about the presence of invasive species may lead to their underestimation or even failure in detecting them due to misidentification of specimens or surveillance strategies that disregard their habitats. In our opinion, more research should be directed to deal with this increasing threat.
